# Supplementary material for: Quantitative macromolecular proton fraction imaging using pulsed spin‐lock
Source: Magn Reson Med. 2025 Aug 5;94(6):2492–507. doi: 10.1002/mrm.70021 (PMC12501701; doi:10.1002/mrm.70021)
Supplement: Supplementary file 1 — Data S1. Supporting Information. [file MRM-94-2492-s001.pdf]

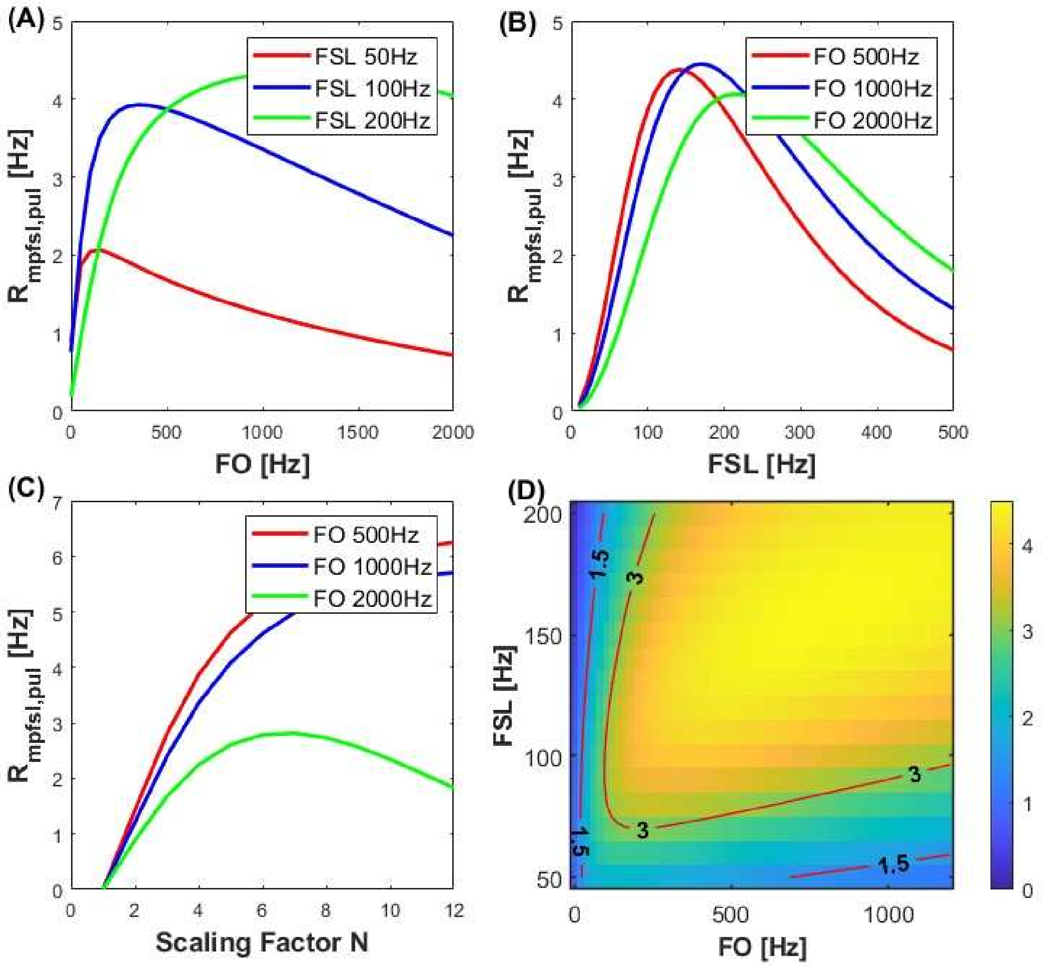

Figure S 1 Relationship between $R_{\mathrm{mpfsl,pul}}$ and the frequency offset (FO) $\Delta\omega^{(1)}$, frequency of spin-lock (FSL) $\omega_1^{(1)}$ and N, where N is a constant scaling factor such that $\Delta\omega^{(2)} = N\Delta\omega^{(1)}$ and $\omega_1^{(2)} = N\omega_1^{(1)}$. A, The $R_{\mathrm{mpfsl,pul}}$ signal as a function of FO, while fixing N = 4 and FSL = 50 Hz, 100 Hz, and 200 Hz, respectively. B, The $R_{\mathrm{mpfsl,pul}}$ signal as a function of FSL, while fixing N = 4 and FO = 500 Hz, 1000 Hz, and 2000 Hz, respectively. C, The $R_{\mathrm{mpfsl,pul}}$ signal as a function of N, while fixing FSL = 100 Hz and FO = 500 Hz, 1000 Hz, and 2000 Hz, respectively. D, The $R_{\mathrm{mpfsl,pul}}$ signal as a function of FO and FSL, while fixing N = 4.

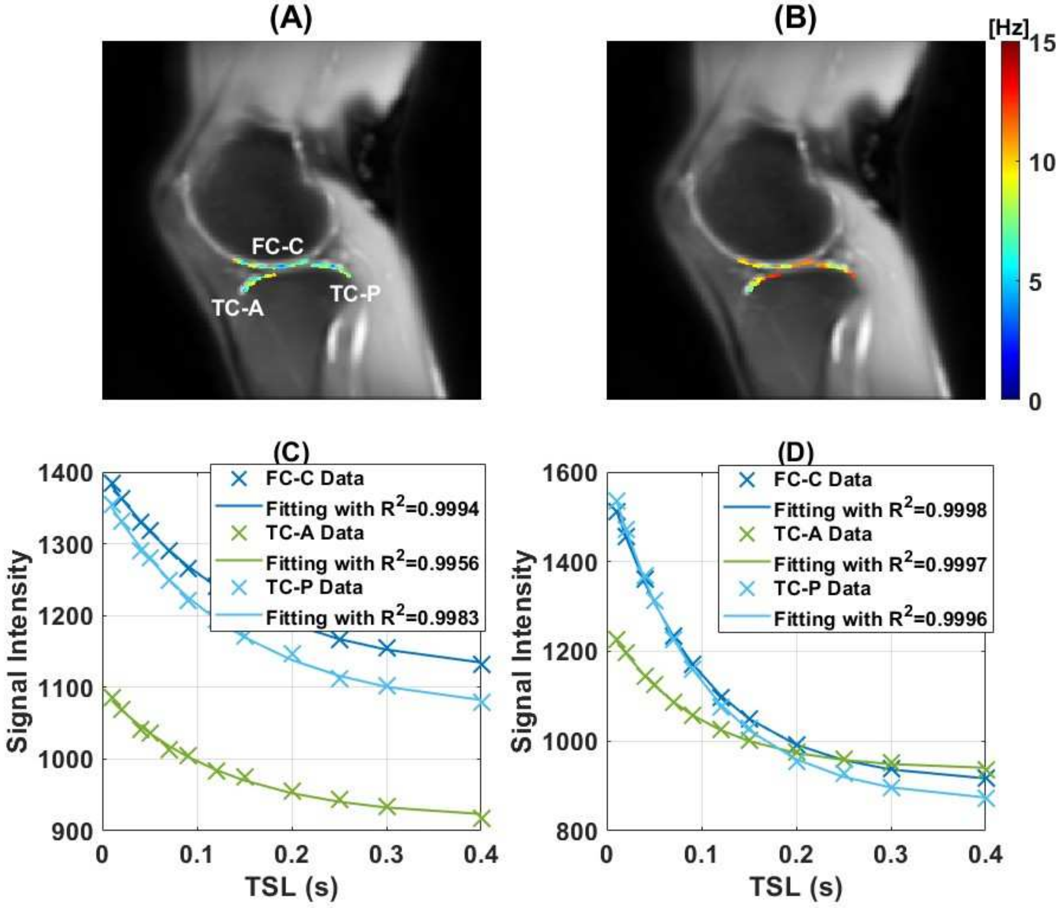

Figure S 2 Off-resonance pulsed $R_{1\rho}$ knee map for a volunteer. (A) Low RF pulse setting: $\omega_1 = 80 \cdot 2\pi$ rad and $\Delta\omega = 800 \cdot 2\pi$ rad. (B) High RF pulse setting: $\omega_1 = 350 \cdot 2\pi$ rad and $\Delta\omega = 3500 \cdot 2\pi$ rad. ROIs FC-C, TC-A, TC-P correspond to the central regions of the femoral cartilage, and the anterior and posterior regions of the tibial cartilage, respectively; (C, D) The low- and high-RF cartilage signals from the ROIs were separately fitted to a mono-exponential model, with the goodness-of-fit metric (R^2) embedded in the legend for quantitative assessment. The background images are the magnitude of the original spin-lock prepared images acquired with the high RF pulse setting.

Derivation S1: The expression of R_{mt}

The longitudinal relaxation rate of MT, R_{mt}, is determined by solving the eigenvalues of the shifted matrix $\mathbf{A}' = \mathbf{A} - \mathrm{diag}(-R_{\mathrm{water}})$[32], under the assumption that the system's eigenvalues are hierarchical. The final expression for R_{mt} is presented in (S1).

$$R_{\mathrm{mt}} = \frac{\left(\Delta\omega^2 + r_{2\mathrm{a}}^2\right)\left(k_{\mathrm{ba}} r_{1\mathrm{a}} + r_{1\mathrm{b}}\left(k_{\mathrm{ba}} + r_{1\mathrm{a}}\right)\right) + \omega_1^2 r_{2\mathrm{a}}^2\left(k_{\mathrm{ba}} + r_{1\mathrm{b}}\right)}{\left(\Delta\omega^2 + r_{2\mathrm{a}}^2\right)\left(k_{\mathrm{ab}} + k_{\mathrm{ba}} + r_{1\mathrm{a}} + r_{1\mathrm{b}}\right) + 2r_{2\mathrm{a}}\left(k_{\mathrm{ba}} r_{1\mathrm{a}} + r_{1\mathrm{b}}\left(k_{\mathrm{ab}} + r_{1\mathrm{b}}\right)\right) + \omega_1^2\left(r_{2\mathrm{a}} + k_{\mathrm{ba}} + r_{1\mathrm{b}}\right)}. \tag{S1}$$

Derivation S2: Transient state relationship with $M_{\mathrm{za}}(t)$ and $M_{\mathrm{zb}}(t)$

In this section, we aim to derive the relationship between $M_{\mathrm{za}}(t)$ and $M_{\mathrm{zb}}(t)$ based on the numerical solutions of Bloch-McConnell equation, as shown in Eq. (S2). The numerical solution of $\mathbf{M(t)} = [M_{\mathrm{xa}}(t), M_{\mathrm{ya}}(t), M_{\mathrm{za}}(t), M_{\mathrm{zb}}(t)]^T$ can be computed using MATLAB's built-in functions for matrix left division (\) and matrix exponentiation (`expm`) as follows,

$$\mathbf{M(t)} = \exp\left(\mathbf{A}t\right) \cdot \left(\mathbf{M}_{\mathrm{ini}} + \mathbf{A^{-1}C}\right) - \mathbf{A^{-1}C}, \tag{S2}$$

where

$$\mathbf{A} = \begin{pmatrix} -R_{2\mathrm{a}} & \Delta\omega & 0 & 0 \\ -\Delta\omega & -R_{2\mathrm{a}} & \omega_1 & 0 \\ 0 & -\omega_1 & -R_{1\mathrm{a}} - k_{\mathrm{ab}} & k_{\mathrm{ba}} \\ 0 & 0 & k_{\mathrm{ab}} & -R_{1\mathrm{b}} - R_{\mathrm{rfb}} - k_{\mathrm{ba}} \end{pmatrix},$$

and

$$\mathbf{C} = \left(0, 0, R_{1\mathrm{a}} M_{0\mathrm{a}}, R_{1\mathrm{b}} M_{0\mathrm{b}}\right)^{\top}. \tag{S3}$$

To begin with, the matrix $\mathbf{A}$ can be diagonalized as $\mathbf{A} = \mathbf{VDU}$, where $\mathbf{D}$ is the diagonal matrix with its diagonal elements representing the eigenvalues of $\mathbf{A}$. The normalization condition $\mathbf{UV} = \mathbf{I}$ always holds. Using this diagonalization, Eq. (S2) can be rewritten as:

$$\mathbf{M}(t) = \mathbf{V}\exp\left(\mathbf{D}t\right)\mathbf{U} \cdot \left(\mathbf{M}_{\mathrm{ini}} + \mathbf{A^{-1}C}\right) - \mathbf{A^{-1}C}, \tag{S4}$$

where u_{ij} is the Matrix element of $\mathbf{U}$, v_{ij} is the matrix element of $\mathbf{V}$.

The solutions of $\mathbf{M}(t)$ can be expressed as combinations of the matrix elements. For instance, $M_{\mathrm{za}}(t)$ can be represented in the following form:

$$M_{\mathrm{za}}(t) = \sum_{j=1}^{4} v_{31} u_{1j} m_j \mathrm{e}^{\lambda_1 t} + \sum_{j=1}^{4} v_{32} u_{2j} m_j \mathrm{e}^{\lambda_2 t} + \sum_{j=1}^{4} v_{33} u_{3j} m_j \mathrm{e}^{\lambda_3 t} + \sum_{j=1}^{4} v_{34} u_{4j} m_j \mathrm{e}^{\lambda_4 t} + \left[\left(\exp\left(\mathbf{A}t\right) - \mathbf{I}\right) \cdot \mathbf{A}^{-1}\mathbf{C}\right]_3, \tag{S5}$$

where $\lambda_{1,2,3,4}$ denote the four eigenvalues of matrix $\mathbf{A}$, while m_{j} correspond to the j-th element of the initial magnetization $\mathbf{M}_{\mathrm{ini}}$. The subscript 3 represents the third element of the corresponding vector.

To derive the relationship between $M_{\mathrm{za}}(t)$ and $M_{\mathrm{zb}}(t)$, it is essential to first understand their individual evolutions. Both $M_{\mathrm{za}}(t)$ and $M_{\mathrm{zb}}(t)$ are characterized by four decay components, corresponding to the eigenvalues of the system. However, the contributions of these components differ for $M_{\mathrm{za}}(t)$ and $M_{\mathrm{zb}}(t)$. As shown in Fig. 1(A), the decay of $M_{\mathrm{za}}(t)$ is dominated by the largest negative real eigenvalue, $R_{1\rho}$ (λ_3), resulting in a mono-exponential decay. The contributions from the other three eigenvalues—one negative real eigenvalue, $R_{1,\mathrm{fast}}$ (λ_4), and two complex eigenvalues, $R_{2\rho} \pm \mathrm{i}\sqrt{\Delta\omega^2 + \omega_1^2}$ ($\lambda_{1,2}$)—are relatively minor and can be neglected for simplicity. In contrast, as illustrated in Fig. 1(B), the decay of $M_{\mathrm{zb}}(t)$ involves comparable contributions from two eigenvalues: $R_{1\rho}$ and $R_{1,\mathrm{fast}}$, leading to a bi-exponential decay profile. By considering only the significant components of $M_{\mathrm{za}}(t)$ and $M_{\mathrm{zb}}(t)$, the following relationship can be derived,

$$M_{\mathrm{zb}}(t)/M_{\mathrm{za}}(t) = \frac{v_{43} u_{33} m_3 \cdot \mathrm{e}^{-R_{1\rho} t} + \left(v_{44} u_{43} m_3 + v_{44} u_{44} m_4\right) \cdot \mathrm{e}^{-R_{1,\mathrm{fast}} t}}{v_{33} u_{33} m_3 \cdot \mathrm{e}^{-R_{1\rho} t}}. \tag{S6}$$

Under the conditions $\Delta\omega \gg \omega_1$ and $R_{1\mathrm{a}} = R_{1\mathrm{b}}$, $R_{1,\mathrm{fast}}$ can be approximated as $k_{\mathrm{ba}} + R_{\mathrm{rfb}}$ and the ratio $v_{33}u_{33}/v_{44}u_{44} \approx 1$. Additionally, the initial condition can be approximated as $m_4 = f_{\mathrm{b}} m_3$. Aside from these, the

remaining relationships between the relevant elements can be derived as follows. Since $\mathbf{V}$ is the left eigenvector matrix of $\mathbf{A}$, these relationships are dictated by the eigenstructure of the system.

$$k_{\mathrm{ab}} v_{33} - \left(R_{1\mathrm{b}} + R_{\mathrm{rfb}} + k_{\mathrm{ba}}\right) v_{43} = \lambda_3 v_{43}. \tag{S7}$$

Under the assumption that $R_{\mathrm{rfb}} + k_{\mathrm{ba}}$ is far larger than $R_{1\mathrm{b}}$ and $R_{1\rho}$, the following relationship between v_{43} and v_{33} can be obtained,

$$v_{43} \approx \frac{f_{\mathrm{b}} k_{\mathrm{ba}}}{R_{\mathrm{rfb}} + k_{\mathrm{ba}}} v_{33}. \tag{S8}$$

The exact relationship between u_{43} and u_{44} is given by: $u_{43}/u_{44} = \left(R_{1\mathrm{b}} + R_{\mathrm{rfb}} + k_{\mathrm{ba}} - R_{1,\mathrm{fast}}\right)/k_{\mathrm{ba}}$. This relationship arises from the fact that $\mathbf{U}$ is the left eigenvector matrix of $\mathbf{A}$. However, for the sake of simplification, we approximate the ratio as: $u_{43}/u_{44} = -k_{\mathrm{ab}}/\left(k_{\mathrm{ba}} + R_{\mathrm{rfb}}\right)$. This approximation incurs only a negligible error while substantially simplifying the formulation of Eq. (S6), making it more convenient for both analytical derivations and practical applications.

Finally, we derived all the relevant element relationships. By incorporating these into Eq. (S6) and assuming $R_{1\rho} \ll R_{1,\mathrm{fast}}$, we obtain the following transient-state relationship between $M_{\mathrm{za}}(\mathrm{t})$ and $M_{\mathrm{zb}}(\mathrm{t})$,

$$M_{\mathrm{zb}}(t)/M_{\mathrm{za}}(t) = f_{\mathrm{b}}(1-\beta) = f_{\mathrm{b}}\left(\frac{k_{\mathrm{ba}}}{k_{\mathrm{ba}} + R_{\mathrm{rfb}}} + \left(\frac{R_{\mathrm{rfb}}}{k_{\mathrm{ba}} + R_{\mathrm{rfb}}}\right) \mathrm{e}^{-\left(k_{\mathrm{ba}} + R_{\mathrm{rfb}}\right)t}\right) \tag{S9}$$

with

$$\beta = \frac{R_{\mathrm{rfb}}}{k_{\mathrm{ba}} + R_{\mathrm{rfb}}}\left(1 - \mathrm{e}^{-\left(k_{\mathrm{ba}} + R_{\mathrm{rfb}}\right)t}\right).$$

The validation of this equation is presented in Fig. 1. The Eq.(6) (also referred to as Eq. (S9)) agrees well numerical solution. Specifically, the 'Reduced form of Eq.(6)' indicated in the legend represents a reduced form of Eq.(6), where the $v_{33} u_{43} m_3 \cdot \mathrm{e}^{-R_{1,\mathrm{fast}} t}$ term is omitted, resulting in a noticeable deviation. This comparison highlights the importance of considering the u_{43}/u_{44} ratio and validates the effectiveness of our approximation for this ratio.

Derivation S3: R_{mpfsl} increases with TSL

In this section, we analyze the cause of the time-dependent behavior of R_{mpfsl}. The underlying reason is that, while magnetization is simplified as a single-exponential decay with constant coefficients (Eq.(2)), the actual situation is far more complex due to the entanglement of various parameters (see Eq. (S5)). We then demonstrate that, when TSL is sufficiently long, the error introduced by the assumption of a constant-coefficient single-exponential model vanishes, and R_{mpfsl} converges to a constant value.

S3.1 | Multi-exponential error

As shown in Fig. 1(A) and Eq. (S5), the longitudinal magnetization dynamics of the water pool during the spin-lock stages do not follow a purely mono-exponential decay. Instead, additional multi-exponential decay components are present, although their contributions are relatively minor. These additional decay terms introduce errors when estimating R_{mpfsl} using Eq. (2), resulting in the time dependence behavior of R_{mpfsl}.

Eigenvalue analysis reveals that the additional multi-exponential terms decay at much faster rates than $R_{1\rho}$. As a result, their influence decreases with increasing total spin-lock duration, eventually becoming negligible.

S3.2 | Amplitude error

The second source of error comes from the sequence-parameters-related component amplitude of the $R_{1\rho}$ decay term. This amplitude depends not only on the initial magnetization in the z-direction but also on the sequence parameters. Consequently, for two acquisitions with different sequence parameters, the amplitudes will differ, ultimately leading to the time dependence of R_{mpfsl}.

To begin, we provide the explicit expression for the amplitude $v_{33} \sum_{j=1}^{4} u_{3\mathrm{j}} m_{\mathrm{j}}$ to demonstrate its relationship with the sequence parameters. As shown in a previous study[31], the partial eigenvector $(v_{13}, v_{23}, v_{33})^{\top}$ can be

approximated using a Taylor expansion as $(\sin\theta, 0, \cos\theta)^\top$. Furthermore, v_{33} and v_{43} are related as described in Eq. (S8). Consequently, the full eigenvector $v_{\cdot 3}$ can be expressed as follows:

$$\mathbf{v}_{\cdot 3}{}^\top = [\sin\theta, 0, \cos\theta, \frac{f_\mathrm{b} k_{ba}}{R_\mathrm{rfb} + k_{ba}} \cos\theta]. \tag{S10}$$

Since the upper-left 3×3 submatrix of $\mathbf{A}$ is Hermitian, the components of the right eigenvector u_{31}, u_{32}, and u_{33} share the same relationship as v_{13}, v_{23}, and v_{33}. Additionally, u_{34} can be expressed as $u_{34} = k_\mathrm{ba} u_{33} / (k_\mathrm{ba} + R_\mathrm{rfb})$, derived using a similar analysis. The full eigenvector $u_{3\cdot}$ with a scalar factor s can thus be expressed as:

$$\mathbf{u}_{3\cdot} = [s\sin\theta, 0, s\cos\theta, s\frac{k_\mathrm{ba}}{R_\mathrm{rfb} + k_\mathrm{ba}} \cos\theta]. \tag{S11}$$

The scalar factor s can be determined by using the normalization condition $\mathbf{uv}^\top = 1$. Consequently, s can be expressed as:

$$s = 1 / \left(1 + \frac{f_\mathrm{b} k_\mathrm{ba}^2}{(R_\mathrm{rfb} + k_\mathrm{ba})^2} \cdot \cos^2\theta\right). \tag{S12}$$

Because $k_\mathrm{ba}/(R_\mathrm{rfb} + k_\mathrm{ba})$ is less than 1, and assuming $f_b \ll 1$, a Taylor expansion can be applied as follows:

$$s = 1 - \frac{k_\mathrm{ab} k_\mathrm{ba}}{(R_\mathrm{rfb} + k_\mathrm{ba})^2} \cdot \cos^2\theta. \tag{S13}$$

Finally, the amplitude of $R_{1\rho}$ decay term is formulated as follows,

$$v_{33} \sum_{j=1}^{4} u_{3\mathrm{j}} m_\mathrm{j} = s\cos\theta \left(\sin\theta m_1 + \cos\theta m_3 + \frac{k_{ab} m_4}{R_\mathrm{rfb} + k_{ba}}\right) \tag{S14}$$

From this equation, it is evident that the amplitude of the mono-exponential decay term explicitly and strongly related to R_rfb, which is affected by the parameters $\Delta\omega$ and ω_1 from two acquisitions. In this case, if we calculate R_mpfsl using Eq. (16),

$$R_\mathrm{mpfsl} = -\log\left(\frac{A^{(2)} \mathrm{e}^{-R_{1\rho}^{(2)}}}{A^{(1)} \mathrm{e}^{-R_{1\rho}^{(1)}}}\right) / \mathrm{TSL} = R_{1\rho}^{(2)} - R_{1\rho}^{(1)} - \log\left(\frac{A^{(2)}}{A^{(1)}}\right) / \mathrm{TSL}. \tag{S15}$$

where $A^{(1)}$ and $A^{(2)}$ are the amplitudes of two acquisitions, which differ slightly due to changes in the sequence parameters. This difference causes R_mpfsl to exhibit time-dependent behavior. As TSL increases, this dependence diminishes, and R_mpfsl converges to a constant value.

Table S1: T_{1a} values of the phantom with and without $MnCl_2$

Agarose	1%	2%	3%	4%
No $MnCl_2$	1541.42 ± 18.21 ms	1680.90 ± 19.26 ms	1832.52 ± 17.91 ms	1740.24 ± 30.00 ms
0.05 mM $MnCl_2$	951.35 ± 29.28 ms	779.51 ± 31.58 ms	833.80 ± 27.61 ms	757.79 ± 56.38 ms

Table S2: T_{2a} values of the phantom with and without $MnCl_2$

Agarose	1%	2%	3%	4%
no $MnCl_2$	127.35 ± 6.76 ms	82.67 ±4.14 ms	59.18 ± 3.40	45.25± 3.66 ms
0.05mM $MnCl_2$	92.89± 3.51ms	58.67± 2.29ms	46.62± 2.75ms	34.95± 2.56ms
